# Sclerosing angiomatoid nodular transformation of the spleen: Case reports and literature review

**DOI:** 10.1097/MD.0000000000038466

**Published:** 2024-06-07

**Authors:** Leizhou Xia, Zhitao Li, Pengcheng Jiang, Yongjun Zhang, Xuefeng Bu, Nana Meng

**Affiliations:** a Department of General Surgery, Affiliated People’s Hospital, Jiangsu University, Zhenjiang, Jiangsu Province, China; b Department of Ophthalmology, Affiliated People’s Hospital, Jiangsu University, Zhenjiang, Jiangsu Province, China; c Zhenjiang Kangfu Eye Hospital, Zhenjiang, Jiangsu Province, China.

**Keywords:** diagnosis, laparoscopic splenectomy, sclerosing angiomatoid nodular transformation, spleen, treatment

## Abstract

**Rationale:**

Sclerosing angiomatoid nodular transformation (SANT) of the spleen is an uncommon benign vascular lesion with an obscure etiology. It predominantly affects middle-aged women and presents with nonspecific clinical signs, making preoperative diagnosis challenging. The definitive diagnosis of SANT relies on pathological examination following splenectomy. This study aims to contribute to the understanding of SANT by presenting a case series and reviewing the literature to highlight the clinical presentation, diagnostic challenges, and treatment outcomes.

**Patient concerns:**

In this retrospective study, we analyzed the clinical data of 3 patients with confirmed SANT admitted from November 2013 to October 2023. The cases include a 25-year-old male, a 15-year-old female, and a 39-year-old male, each with a splenic mass.

**Diagnoses and Interventions:**

All of the three cases were treated by laparoscopic splenectomy (LS). Pathological examination confirmed SANT in all cases.

**Outcomes:**

No recurrence or metastasis was observed during a 10-year follow-up for the first 2 cases, and the third case showed no abnormalities at 2 months postoperatively. Despite its rarity, SANT is a significant condition due to its potential for misdiagnosis and the importance of distinguishing it from malignant lesions. The study underscores the utility of LS as a safe and effective treatment option.

**Lessons:**

SANT is a rare benign tumor of the spleen, and the preoperative diagnosis of whom is challenging. LS is a safe and effective treatment for SANT, with satisfactory surgical outcomes and favorable long-term prognosis on follow-up. The study contributes to the limited body of research on this rare condition and calls for larger studies to validate these findings and improve clinical management.

## 1. Introduction

Sclerosing angiomatoid nodular transformation of the spleen (SANT) is an infrequent benign vascular neoplasm that accounts for approximately 1.68% of benign splenic lesions.^[1]^ The term SANT was first proposed by Martel et al in 2004, who described the pathological concept of this condition.^[2]^ The precise etiology of SANT remains elusive, and it typically affects middle-aged individuals between 30 and 60 years, with a higher prevalence in females. Due to its rarity and nonspecific clinical symptoms, preoperative diagnosis of SANT is often challenging.^[3]^ In this study, we retrospectively analyzed the clinical data of 3 patients with confirmed SANT, who were admitted to Affiliated People Hospital, Jiangsu University from November 2013 to October 2023. The details are reported as follows:

## 2. Case presentation

### 2.1. Case 1

A 25-year-old male patient presented with an incidental splenic mass during a routine examination. Physical examination and laboratory tests were unremarkable. Abdominal computerized tomography (CT) revealed a well-defined, enhancing mass measuring 77 mm*84 mm. The patient underwent laparoscopic splenectomy (LS) during which a firm spherical mass measuring approximately 7 cm in diameter was discovered in the lower pole of the spleen, with intact capsule. The surgery lasted 170 minutes, and the estimated intraoperative blood loss was around 50 ml. Pathological examination confirmed SANT of the spleen, with positive staining for CD8, CD31, and CD34, while SMA, CD21, CD23, and Ki67 were negative. The patient remained disease-free during the 10-year follow-up period, with no recurrence or metastasis.

### 2.2. Case 2

A 15-year-old female presented with a history of chest tightness and exertional dyspnea for 3 years, along with the incidental finding of a splenic mass for over a month. Physical examination revealed enlarged superficial neck lymph nodes, whose biopsy indicated reactive proliferation without malignant changes. Cardiovascular and respiratory examinations were unremarkable. Abdominal examination showed no palpable spleen. CA50 level was 46.90 U/ml, and other laboratory tests were within normal limits. Abdominal CT revealed an enlarged spleen with an irregularly enhanced mass measuring approximately 97 mm*79 mm. The patient underwent LS, during which a spherical mass measuring approximately 8 cm in diameter was found in the upper pole of the spleen, with intact capsule. The procedure lasted 155 minutes, and the estimated intraoperative blood loss was around 30 ml. Pathological examination confirmed the diagnosis of SANT of the spleen, supported by positive immunohistochemistry staining for CD31, CD34, CD68, and Ki67 (5%), as well as SMA and CD8. The patient remained disease-free during the 10-year follow-up period, with no abnormalities detected in hematological examinations or CT scans.

### 2.3. Case 3

**Figure 1.**
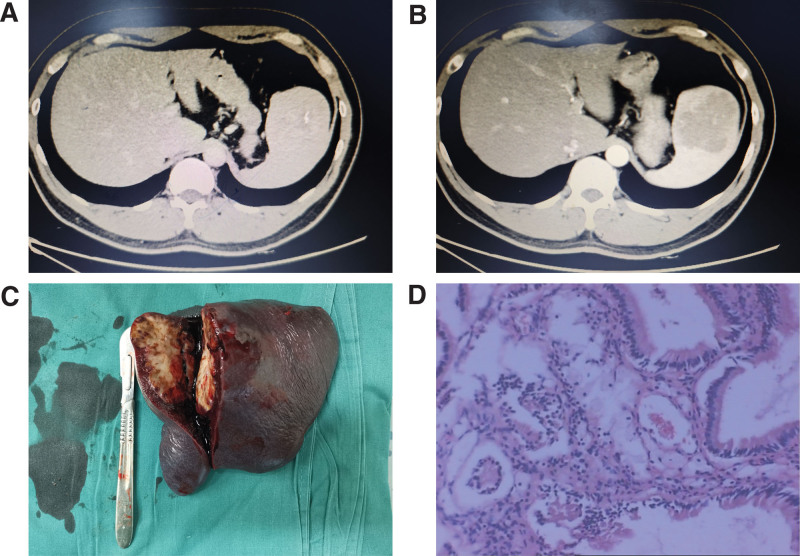
The representative CT scanning photos, resected specimen of the spleen and pathological findings of case 3. (A) Abdominal computed tomography (CT) scan showed spleen lesions characterized by elliptical low-density areas accompanied by calcification. (B) Enhanced abdominal CT scan indicated peripheral enhancement of the spleen during the arterial phase. (C) The resected spleen specimen displayed an approximately 5 cm diameter mass located in the upper pole of the spleen. (D) The pathological examination revealed sclerosing angiomatoid nodular transformation of the spleen (Hematoxylin & Eosin staining, ×100).

A 39-year-old man presented with recurrent left upper abdominal pain for 6 months, along with concurrent gallbladder stones and chronic cholecystitis. Physical examination and laboratory tests did not reveal any significant abnormalities. Abdominal CT revealed enlargement and elliptical low-density areas with linear calcification at the splenic edge and contrast enhancement, measuring approximately 57 mm (Fig. 1A and 1B). The patient underwent LS along with laparoscopic cholecystectomy. Intraoperative exploration revealed a firm spherical mass measuring approximately 5 cm in diameter in the upper pole of the spleen, with intact capsule (Fig. 1C). The surgery lasted 170 minutes, and the estimated intraoperative blood loss was around 20 mL. Pathological examination confirmed the diagnosis of SANT of the spleen (Fig. 1D). Follow-up at 3 months postoperatively showed no abnormalities in hematological examinations. Imaging follow-up had not been conducted at the time of this report.

### 2.4. Outcomes

The study presents a retrospective analysis of 3 cases of SANT of the spleen, focusing on clinical presentation, imaging examinations, treatment, and follow-up outcomes. All 3 patients underwent LS as the primary treatment for their SANT, with average surgery duration and blood loss being 155 minutes and 33.3 mL, respectively. The surgical procedures were uneventful, and postoperative recovery was satisfactory in all cases. The first 2 cases were temporarily administered aspirin due to transient thrombocytosis postoperatively. During the 10-year follow-up period for the first 2 cases, there were no reports of recurrence or metastasis. For the third case, no abnormalities were reported at the 3-month postoperative follow-up.

## 3. Discussion

SANT is a benign tumor of the spleen characterized by red marrow vascular proliferation and the formation of multiple sclerotic nodules.^[3]^ However, the exact pathogenesis of SANT is still not fully understood. Some researchers believe that SANT may represent the end stage of benign splenic lesions such as capillary hemangioma or hamartoma.^[4]^ Other studies suggest a possible association between SANT and EB virus infection, as well as autoimmune diseases related to IgG4.^[5,6]^

SANT is commonly seen in middle-aged women, although there have been reported cases in children as well.^[7]^ As the number of reported cases and research increases, the incidence rate in women has decreased.^[8]^ Our study has a small sample size and is a single-center study, including only 3 cases of SANT, with 2 males and 1 female, and the average age being only 26.3 years, which differs from the reported results in the literature.

Clinically, most patients with SANT are asymptomatic and do not exhibit splenic hyperfunction. Some patients may experience abdominal or back pain, as well as abnormal hematological findings.^[3,4]^ Therefore, preoperative diagnosis of SANT remains challenging. In our study, 1 case was asymptomatic with an incidental finding of splenic mass on physical examination, 1 case had associated with enlarged superficial neck lymph nodes and slightly elevated CA50, and 1 case had left upper abdominal pain, consistent with previous reports.

Imaging examinations, such as CT and magnetic resonance imaging, have some value in diagnosing SANT; however, they cannot differentiate it from other types of vascular lesions. CT scans typically show a single, round, low-density mass with clear borders and possible calcifications. Contrast-enhanced scans may reveal nodular enhancement or a “spoke-wheel” pattern. On MR-enhanced scans, typical centripetal and progressive enhancement can be observed.^[9,10]^ In our study, MR scans were not performed in the 3 cases of SANT, but the CT findings were consistent with previous reports. However, these features are not specific to SANT and may resemble other vascular lesions or even malignant tumors, such as angiosarcoma. Therefore, histopathological examination remains the gold standard for diagnosing this rare condition.^[3,11]^ Immunohistochemical analysis has shown high expression rates of CD34, CD31, CD8, CD68, and SMA in SANT nodules, which has a high diagnostic value.^[3]^ In our study, 2 cases underwent immunohistochemical examination, with results consistent with previous reports.

Splenectomy is the gold standard treatment for SANT, and in recent years, laparoscopic surgery has become a more reliable method for its management.^[12]^ SANT is a benign splenic condition with a good prognosis. To date, there have been no reports of recurrence following (laparoscopic) splenectomy.^[13]^ During the operation, it is important to ensure complete removal of the spleen to avoid potential implantation metastasis of malignant lesions. In the present study, all 3 cases of SANT underwent LS, with uneventful surgical procedures and postoperative recovery.

The current study, while providing valuable insights into the clinical presentation and management of SANT of the spleen, has its limitations. The study includes only 3 cases of SANT, which limits the statistical power to draw definitive conclusions. The small sample size also restricts the ability to perform subgroup analyses, which could reveal important differences in disease presentation or treatment outcomes. The cases were all treated at a single medical center, which may limit the diversity of patient characteristics and treatment approaches. Moreover, the study does not provide new insights into the etiology of SANT, which remains a significant knowledge gap. The lack of understanding of the underlying cause of the condition limits the ability to develop targeted preventive or therapeutic strategies. Further research with a larger sample size and a comprehensive analysis of literatures is indispensable to validate the results and to better understand the epidemiology, clinical spectrum, and optimal management of SANT.

## 4. Conclusion

In conclusion, SANT is a rare benign tumor of the spleen. To date, there have been fewer than 200 reported cases in the English literature and <400 reported cases in Chinese literature.^[3]^ Preoperative diagnosis of SANT remains challenging, and histopathological examination is essential for accurate diagnosis. Laparoscopic splenectomy is a safe and effective treatment option for SANT, resulting in favorable surgical outcomes and long-term prognoses. However, further research is needed to enhance our understanding of this rare condition.

## Acknowledgments

The authors thank the studies and books which were included in this article.

## Author contributions

**Conceptualization:** Leizhou Xia, Zhitao Li, Pengcheng Jiang, Yongjun Zhang, Xuefeng Bu, Nana Meng.

**Funding acquisition:** Leizhou Xia, Nana Meng.

**Investigation:** Zhitao Li.

**Resources:** Yongjun Zhang, Xuefeng Bu.

**Writing – original draft:** Leizhou Xia, Zhitao Li, Pengcheng Jiang, Xuefeng Bu, Nana Meng.

**Writing – review & editing:** Leizhou Xia, Nana Meng.
